# Self‐reported visual difficulties in Europe and related factors: a European population‐based cross‐sectional survey

**DOI:** 10.1111/aos.14643

**Published:** 2020-10-07

**Authors:** Nicolas Leveziel, Simon Marillet, Tasanee Braithwaite, Tunde Peto, Pierre Ingrand, Shahina Pardhan, Alain M. Bron, Jost B. Jonas, Serge Resnikoff, Julie‐Anne Little, Rupert R.A. Bourne

**Affiliations:** ^1^ Vision & Eye Research Institute Anglia Ruskin University Cambridge UK; ^2^ CHU Poitiers Poitiers France; ^3^ CIC 1402 Poitiers France; ^4^ INSERM 1084 Poitiers France; ^5^ University of Poitiers Poitiers France; ^6^ Centre for Patient Reported Outcomes Research and NIHR Birmingham Biomedical Research Centre University of Birmingham Birmingham UK; ^7^ Moorfields Eye Hospital London UK; ^8^ Institute of Clinical Sciences Building A Queen's University Belfast Belfast UK; ^9^ Epidemiology and biostatistics department Faculty of Medicine University of Poitiers Poitiers France; ^10^ Department of Ophthalmology University Hospital Dijon France; ^11^ Eye and Nutrition Research Group Bourgogne Franche‐Comté University Dijon France; ^12^ Department of Ophthalmology Medical Faculty Mannheim Heidelberg University Mannheim Germany; ^13^ Brien Holden Vision Institute and SOVS University of New South Wales Sydney NSW Australia; ^14^ Centre for Optometry & Vision Science Biomedical Sciences Ulster University Coleraine UK; ^15^ Cambridge Eye Research Centre Department of Ophthalmology Cambridge University Hospitals Cambridge UK

**Keywords:** associated factors, epidemiology, Europe, ophthalmology, prevalence, vision impairment, vision loss

## Abstract

**Purpose:**

There is a relative paucity of self‐reported vision problems data in European countries.

**Methods:**

In this context, we investigated self‐reported vision problems through European Health Interview Survey 2, a cross‐sectional European population survey based on a standardized questionnaire including 147 medical, demographic and socioeconomic variables applied to non‐institutionalized individuals aged 15 years or more in 28 European countries, in addition to Iceland and Norway.

**Results:**

The survey included 311 386 individuals (54.18% women), with overall crude prevalence of self‐reported vision problems of 2.07% [95% CI; 2.01–2.14]. Among them, 1.70 % [1.61–1.78] of men, 2.41% [2.31–2.51] of women and 4.71% [4.53–4.89] of individuals aged 60 or more reported to have a lot of vision problems or to be not able to see. The frequency of self‐reported vision problems was the highest in Eastern European countries with values of 2.43% [2.30–2.56]. In multivariate analyses, limiting long‐standing illness, depression, daily smoking, lack of physical activity, lower educational level and social isolation were associated with self‐reported vision problems with ORs of 2.66 [2.42–2.92], 2.16 [2.01–2.32], 1.11 [1.01–1.23], 1.31 [1.21–1.42], 1.29 [1.19–1.40] and 1.45 [1.26–1.67], respectively, while higher income was associated with less self‐reported vision problems with OR of 0.80 [0.73–0.86].

**Conclusions:**

This study demonstrated inequalities in terms of prevalence of self‐reported vision problems in Europe, with higher prevalence in Eastern European countries and among women and older individuals.

## Introduction

In addition to reducing educational and economic opportunities, blindness and visual impairment have been linked to lower quality of life, shorter life expectancy and higher morbidity (McCarty et al. 2001; Lee et al. 2002; Thiagarajan et al. 2005; Knudtson et al. 2006; Cugati et al. 2007; Karpa et al. 2009; Chakravarthy et al. 2017; Wang et al. 2017). Identification of factors that link vision problems with morbidity and premature death can assist with prevention and improve welfare of those with existing vision impairment.

In 2017, the Global Burden of Disease Vision Loss Expert Group published a population‐based prevalence study of visual impairment and blindness worldwide, followed by a paper focussing on prevalence and causes of vision loss in high‐income countries and in Eastern and Central Europe (Bourne et al. 2017; Bourne et al. 2018). In these comprehensive systematic reviews covering a twenty‐five‐year period, the authors highlighted the paucity of data from Central and Eastern European countries. The European Health Interview Survey (EHIS 2), a European Union initiative, is a general population‐based survey providing cross‐sectional national data on health status, health determinants and healthcare activities in the European Union. In this study, we examined associations between self‐reported vision difficulties in the EHIS 2 and other variables included in the survey and other European socioeconomic variables.

We sought to ascertain the association between self‐reported vision problems and other variables of interest having a potential interaction with vision problems, identified through review of the literature. Specifically, we focused on medical history of diabetes and depression (Cosh et al. 2018; Aljied et al. 2018; Yu et al. 2019; Schubert et al. 2019) and potential associated risk factors including smoking status (Nita et al. 2017a; Nita et al. 2017; Mitchell et al. 2018), gender inequity (Mganga H et al. 2011; Bourne et al. 2017) and social isolation (Brunes et al. 2019).

## Material and Methods

### Study design and population

The study was performed under the auspices of the EUROVISION research programme, funded by the European Union Horizon 2020 in 2018 (H2020‐EU.1.3.2). The EUROVISION project aims to describe the prevalence of self‐reported vision problems in European countries and to identify related demographic and socioeconomic factors, health determinants and healthcare access issues. The European Health Interview Survey (EHIS 2) was performed between 2013 and 2015 and was designed to include population‐based samples representative of the European population aged 15 years and older. People living in collective households or institutions were excluded from this survey. The survey was conducted in 28 member states of the European Union and in two neighbouring countries (Iceland and Norway).

### Procedures

**Fig. 1:**
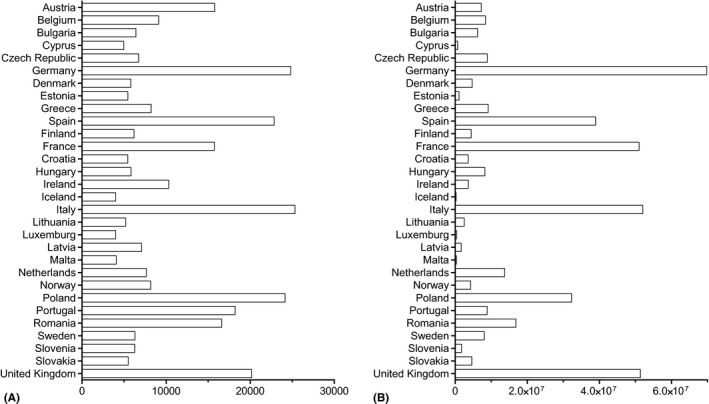
Sample size and total weight for EHIS 2. (A) Sample size (number of respondents). (B) Targeted population (sum of unit weights).

The sampling frame was defined from population census, population registers, dwelling registers, national health insurance registries, postcode address files or samples from the Labour Force Survey, depending on the countries participating in the survey. Using standardized questionnaires, the data were collected by face‐to‐face or telephone interviews, regular mail, email or through the Internet, with the majority of the data originating from telephone and face‐to‐face interviews. Eurostat recommended a minimal required sample size of 7000 individuals per country. This sample size was not reached for member states with a small population (Slovakia, Slovenia, Sweden, Malta, Luxembourg, Lithuania, Iceland, Hungary, Croatia, Finland, Estonia, Denmark, Czech Republic, Cyprus and Belgium). For all these countries, except Malta, Luxembourg and Iceland, the number of respondents was above 5000 (Fig. 1).

The standardized questionnaire included four different modules comprising a demographic and socioeconomic component and public health category divided into a European health status module, a European health determinant module and a European healthcare module (Table 1). The questionnaire included 147 variables in total.

**Table 1:** Composition of public health modules developed into the questionnaires.

European Health Status Module	European Health Determinants Module	European Health Care Module
Health status	Weight and height	Use of inpatient and day care services
Specific diseases & chronic conditions	Physical activity	Use of ambulatory and home care
Occurrence of accidents and injuries	Consumption of fruits and vegetables	Medicine use
Absence from work (health problems)	Smoking behaviour	Use of preventive services
Physical & sensory functional limitations	Alcohol consumption	Unmet needs for health care
Difficulties with personal care activities	Social support	
Difficulties with household activities	Provision of informal care or assistance	
Having pain		
Specific aspects of mental health		

### Categorising variables

From the original age groups, two alternative categorizations have been created. First, in order to account for varying top‐coding across countries, age groups 75–79, 80–84, 80+ and 85+ have been merged in one group (75+). These groups were used for the global and region‐wise univariate analysis, the multivariate analysis and for age standardization. Second, in order to reach a large enough sample size in each group to obtain reliable results for logistic regression within each country, adult individuals have been pooled in the following groups 18–29, 30–39, 40–49, 50–59, 60–69 and 70+. These groups were used for the individual countries univariate analysis.

Two groups pertaining to vision status were defined: ‘no vision problems’ and ‘vision problems’. These groups were derived from the variable named PL2 (‘Difficulty in seeing, even when wearing glasses or contact lenses’). The possible answers were 1: ‘No difficulty’, 2: ‘Some difficulty’, 3: ‘A lot of difficulty’ and 4: ‘Cannot do at all/unable to do’. Individuals who answered 3 or 4 were included in the ‘vision problems’ group. Those who did not answer were excluded. We defined ‘unmet need’ for optical correction as the proportion of respondents within the ‘vision problems’ group who also reported not wearing an optical correction.

### Associated factors

Aside from age and gender, other variables were created to investigate their association with vision difficulties. These variables included education, wealth, health, daily smoking, daily alcohol consumption, physical activity, depression, chronic conditions, functional limitations (for respondents aged 65 years or more), limiting long‐standing illness and social isolation.

Education was categorized into three levels: ‘low’ for preprimary to lower secondary education, ‘intermediate’ for upper secondary to short cycle tertiary education and ‘higher’ for tertiary education and above. Wealth was coded in two levels: ‘low’ for income in the lowest quintile and ‘higher’ for income in the other quintiles. Self‐assessed health was coded in two levels: ‘good’ for respondent who judged their health ‘good’ or ‘very good’, ‘poor’ for those who answered ‘fair’, ‘bad’ or very ‘bad’. Physical activity was coded ‘yes’ if the respondent walked, used a bike, practiced sports, fitness or recreational physical activities for 30 min or more at least once a week, and ‘no’ otherwise. Depression was assessed by either a response confirming depression, or from scoring more than three negative responses out of six items relating to mental well‐being (chosen to be as similar as possible to the Center for Epidemiologic Studies‐Depression scale). Chronic condition indicators included self‐reported diabetes, heart problems (coronary heart disease, angina pectoris or myocardial infarction) and stroke. The chronic condition indicators were combined into a single indicator variable encoding ‘one or more chronic conditions’. Functional limitations were assessed by different items including difficulty walking half a kilometre on level ground, difficulty walking up or down 12 steps, difficulty feeding oneself, difficulty getting in and out of a bed or chair, difficulty dressing and undressing, difficulty using toilets and difficulty in bathing or showering. These items were combined in a single indicator variable encoding ‘one or more functional limitations’. This variable was only defined for respondents aged 65 years and older. Social isolation was assessed by combining the following variables: partnership status and inadequate financial support. Respondents who were single and had inadequate financial support were deemed socially isolated. Respondents were defined as either living as a couple (married or not) or single according to their reported marital and consensual union status. Inadequate financial support was assessed by the inability of respondents to afford medical examination or treatment over the past 12 months. A more detailed definition of these variables is provided in Supporting Information (Table S1.)

### Additional data

In addition to the data collected through the questionnaires, other country‐level socioeconomic indicators relating to each country corresponding to the time of the EHIS survey were included in the analyses. The Human Development Index (HDI), the Gender Inequality Index (GII) and the Inequality adjusted human development index (IHDI) were obtained from the United Nations Development Programme (http://hdr.undp.org/en/indicators/137506). The Gross Domestic Product per capita (GDP), Current Health Expenditure (CHE) and out‐of‐pocket expenditure (% of current health expenditure) were obtained from the World Bank (https://data.worldbank.org/indicator).

Countries participating in the survey were grouped in four European regions defined by the United Nations as follows. Western Europe: Germany, Austria, Belgium, France, Luxembourg and The Netherlands; Eastern Europe: Bulgaria, Hungary, Poland, Czech Republic, Romania and Slovakia; Northern Europe: Norway, Iceland, Ireland, Lithuania, Latvia, UK, Sweden, Finland, Denmark and Estonia; Southern Europe: Croatia, Spain, Greece, Italy, Malta, Portugal and Slovenia. Note that while not part of any region, Cyprus was included in the overall analysis.

### Statistical analysis

All analyses were performed using the survey unit weights supplied within the EHIS 2 data set. These made adjustments to the crude data to enhance the representativeness of the survey data in relation to the sampled national population. According to the survey guidelines, they were specified to allow for overall calculations and inter‐country comparisons, and accounted for sampling design, non‐response, gender and age structure of the populations, and (in some of the datasets) also regional distribution and educational attainment. The SAS procedure surveyfreq was used to compute crude prevalence and associated 95% confidence intervals (CI) taking these weights into account.

Odds ratios and their 95% CI were computed using logistic regression (SAS surveylogistic procedure), adjusting for age and sex. For the univariate analysis, only complete observations for the variable of interest (without missing data) were used. For the multivariate analyses, data imputation was first carried out due to the small proportion of complete observations across all variables of interest (61%) and also to mitigate possible bias due to a few countries not asking some questions.

Age‐adjusted prevalence and 95% CIs were computed using the direct method (SAS stdrate procedure). The reference population was taken to be the 5‐year wide European (28) population data from Eurostat (https://appsso.eurostat.ec.europa.eu/nui/show.do?dataset=demo_pjan&lang=en). The average was taken for the period from 2013 to 2015.

Least‐square linear regression (SAS reg procedure) was used for the regression analyses.

All analyses were performed with SAS/STAT software, version 9.4 of the SAS System for Windows. Copyright © 2016 by SAS Institute Inc. All figures were created using GraphPad Prism version 5.03 for Windows, GraphPad Software, La Jolla California USA.

## Results

EHIS 2 included 316,333 participants of whom 4947 (1.6%) were excluded because of missing vision status data. The analysed sample thus consisted of 311 386 respondents (54.18% women), including 302 093 adults aged 18 or older and 9293 teenagers aged 15–17 years old (2.98%). Age group sizes ranged from 6938 (ages 18–19) to 27 589 (ages 50–54). Of the sample analysed, 55.81% of men and 66.37% of women reported that they wore glasses or contact lenses, and 1.84% of men and 2.91% of women reported vision problems.

The overall crude prevalence of self‐reported vision problems was 2.07% [2.01–2.14]. Among people reporting vision problems in Europe (2.07%), almost a quarter (26%) did not report using optical correction (0.54%). The unmet need for optical correction despite vision problems was 20% in Eastern, 25% in Northern, 30% in Southern and 41% in Western European regions. Considering the analysis by region and country, respondents in Southern and Western European countries showed similar crude prevalence of self‐reported vision problems with values of 2.29% [2.17–2.41] and 2.17% [2.03–2.31], respectively (OR and 95% CI for Western versus Southern country: 1.01 [0.92–1.09]). On the other hand, Eastern and Northern countries, respectively, had the highest and lowest crude prevalence with values of 2.43% [2.30–2.56] and 1.25% [1.14–1.36] (OR and 95% CI for Northern versus Eastern country: 0.49 [0.44–0.54]). The remaining ORs and 95% CI are as follows: Southern versus Eastern: 0.82 [0.76–0.89]; Western versus Eastern: 0.83 [0.76–0.90]; Southern versus Northern: 1.69 [1.52–1.88]; Western versus Northern: 1.70 [1.52–1.91]. Among each region, there were considerable inter‐country differences, ranging from 0.86 [0.66–1.06] and 0.86 [0.59–1.13] in Ireland and Malta, respectively, to 4.31% [3.91–4.70] and 6.48 [5.76–7.19] in Portugal and Belgium, respectively. These data are detailed by region and by country for three age groups (<18, 18–65, ≥60 years old) and by gender in Table 2.

**Table 2:** Crude prevalence (%) of self‐reported vision problems provided by region and by country for three age groups and by sex.

	All	Age			Need for optical correction	
		15–17	18‐59	60+	Met	Unmet
Europe	2.07 [2.01–2.14] N = 311 386	0.49 [0.30–0.68] N = 9293	1.02 [0.96–1.08] N = 194 912	4.71 [4.53–4.89] N = 107 181	1.53 [1.47–1.59] N = 191 603	0.54 [0.51–0.58] N = 119 783
East	2.43 [2.30–2.56] N = 65 182	0.52 [0.21–0.83] N = 2024	0.94 [0.83–1.04] N = 40 257	6.34 [5.96–6.71] N = 22 901	1.94 [1.83–2.06] N = 32 534	0.49 [0.43–0.55] N = 32 648
Bulgaria	2.10 [1.76–2.44] N = 6400	0.00 [0.00–0.00] N = 188	0.43 [0.23–0.64] N = 3829	5.64 [4.68–6.60] N = 2383	1.40 [1.12–1.68] N = 2978	0.70 [0.50–0.90] N = 3422
Czech Republic	2.04 [1.71–2.38] N = 6737	0.00 [0.00–0.00] N = 120	0.88 [0.53–1.22] N = 3408	4.79 [4.00–5.58] N = 3209	1.78 [1.46–2.09] N = 4507	0.27 [0.15–0.38] N = 2230
Hungary	2.63 [2.21–3.06] N = 5825	1.76 [0.00–3.77] N = 204	1.54 [1.15–1.93] N = 3891	5.20 [4.11–6.29] N = 1730	1.79 [1.44–2.15] N = 2899	0.84 [0.60–1.08] N = 2926
Poland	3.17 [2.92–3.41] N = 24 125	0.80 [0.25–1.35] N = 874	1.25 [1.05–1.44] N = 15 390	8.53 [7.80–9.27] N = 7861	2.66 [2.44–2.88] N = 13 343	0.51 [0.41–0.61] N = 10 782
Romania	1.62 [1.42–1.82] N = 16 605	0.00 [0.00–0.00] N = 498	0.39 [0.26–0.51] N = 10 104	4.86 [4.23–5.49] N = 6003	1.18 [1.02–1.35] N = 5702	0.43 [0.33–0.54] N = 10 903
Slovakia	1.10 [0.84–1.35] N = 5490	0.00 [0.00–0.00] N = 140	0.43 [0.23–0.64] N = 3635	3.20 [2.37–4.04] N = 1715	1.00 [0.76–1.25] N = 3105	0.09 [0.02–0.16] N = 2385
North	1.25 [1.14–1.36] N = 76 999	0.48 [0.12–0.83] N = 2203	0.77 [0.65–0.90] N = 45 941	2.50 [2.25–2.75] N = 28 855	0.93 [0.84–1.03] N = 50 878	0.32 [0.26–0.38] N = 26 121
Denmark	1.00 [0.75–1.25] N = 5510	0.50 [0.00–1.48] N = 163	0.67 [0.40–0.95] N = 3169	1.81 [1.23–2.40] N = 2178	0.59 [0.40–0.78] N = 3910	0.41 [0.25–0.57] N = 1600
Estonia	2.25 [1.86–2.65] N = 5449	0.81 [0.00–2.40] N = 185	0.65 [0.39–0.92] N = 3440	6.15 [4.98–7.33] N = 1824	1.68 [1.34–2.02] N = 3364	0.57 [0.36–0.78] N = 2085
Finland	1.92 [1.57–2.27] N = 5982	0.00 [0.00–0.00] N = 178	1.31 [0.91–1.71] N = 3287	3.38 [2.63–4.13] N = 2517	1.59 [1.27–1.91] N = 4446	0.34 [0.19–0.49] N = 1536
Iceland	1.13 [0.80–1.46] N = 3991	0.00 [0.00–0.00] N = 227	0.81 [0.45–1.16] N = 2680	2.37 [1.47–3.27] N = 1084	0.93 [0.64–1.23] N = 2459	0.20 [0.06–0.34] N = 1532
Ireland	0.86 [0.66–1.06] N = 9567	0.00 [0.00–0.00] N = 74	0.59 [0.37–0.82] N = 5986	1.90 [1.42–2.38] N = 3507	0.63 [0.45–0.80] N = 6229	0.23 [0.13–0.33] N = 3338
Latvia	2.69 [2.32–3.06] N = 7068	0.00 [0.00–0.00] N = 241	0.87 [0.57–1.16] N = 4296	7.06 [6.04–8.08] N = 2531	1.47 [1.20–1.74] N = 2892	1.22 [0.96–1.48] N = 4176
Lithuania	2.05 [1.69–2.41] N = 5205	1.02 [0.00–2.43] N = 194	0.81 [0.50–1.12] N = 3139	5.11 [4.12–6.10] N = 1872	1.42 [1.12–1.72] N = 2622	0.63 [0.43–0.83] N = 2583
Norway	0.92 [0.68–1.16] N = 8161	0.65 [0.00–1.61] N = 319	0.62 [0.39–0.85] N = 5467	1.72 [1.05–2.39] N = 2375	0.71 [0.50–0.92] N = 5184	0.21 [0.10–0.33] N = 2977
Sweden	1.60 [1.25–1.95] N = 5939	0.22 [0.00–0.64] N = 274	1.10 [0.77–1.42] N = 4051	2.87 [1.96–3.78] N = 1614	1.13 [0.83–1.42] N = 3868	0.47 [0.28–0.67] N = 2071
United Kingdom	1.11 [0.95–1.27] N = 20 127	0.58 [0.00–1.17] N = 348	0.72 [0.54–0.91] N = 10 426	2.15 [1.80–2.49] N = 9353	0.86 [0.72–0.99] N = 15 904	0.25 [0.16–0.34] N = 4223
South	2.29 [2.17–2.41] N = 89 132	0.46 [0.12–0.80] N = 2406	0.92 [0.82–1.02] N = 54 071	5.47 [5.15–5.78] N = 32 655	1.60 [1.51–1.70] N = 53 854	0.69 [0.62–0.75] N = 35 278
Croatia	2.95 [2.47–3.43] N = 5396	1.48 [0.00–3.29] N = 185	1.12 [0.73–1.51] N = 3272	7.10 [5.80–8.40] N = 1939	2.36 [1.92–2.80] N = 2871	0.59 [0.38–0.80] N = 2525
Greece	2.28 [1.96–2.61] N = 8216	0.00 [0.00–0.00] N = 120	0.63 [0.39–0.87] N = 4734	5.98 [5.08–6.87] N = 3362	1.81 [1.53–2.09] N = 4719	0.47 [0.31–0.63] N = 3497
Italy	2.04 [1.85–2.22] N = 24 256	0.50 [0.00–1.00] N = 793	0.73 [0.58–0.87] N = 15 046	4.77 [4.29–5.25] N = 8417	1.51 [1.35–1.66] N = 13 861	0.53 [0.43–0.62] N = 10 395
Malta	0.86 [0.59–1.13] N = 4045	1.13 [0.00–3.33] N = 109	0.37 [0.14–0.60] N = 2459	2.04 [1.32–2.77] N = 1477	0.65 [0.42–0.89] N = 2715	0.21 [0.07–0.35] N = 1330
Portugal	4.31 [3.91–4.70] N = 18 194	0.22 [0.00–0.58] N = 435	2.41 [2.02–2.80] N = 10 503	8.79 [7.85–9.73] N = 7256	2.79 [2.48–3.10] N = 11 086	1.52 [1.27–1.76] N = 7108
Slovenia	2.50 [2.08–2.93] N = 6195	0.41 [0.00–1.22] N = 243	1.28 [0.89–1.66] N = 3978	5.64 [4.49–6.78] N = 1974	2.03 [1.65–2.41] N = 3723	0.47 [0.28–0.67] N = 2472
Spain	2.10 [1.89–2.31] N = 22 830	0.47 [0.00–1.18] N = 521	0.87 [0.69–1.05] N = 14 079	5.39 [4.81–5.98] N = 8230	1.33 [1.17–1.49] N = 14 879	0.77 [0.64–0.90] N = 7951
West	2.17 [2.03–2.31] N = 75 115	0.51 [0.12–0.90] N = 2439	1.29 [1.16–1.41] N = 51 286	4.45 [4.07–4.83] N = 21 390	1.59 [1.47–1.71] N = 51 591	0.59 [0.51–0.66] N = 23 524
Austria	1.39 [1.09–1.69] N = 15 771	0.00 [0.00–0.00] N = 252	0.74 [0.57–0.91] N = 11 732	3.19 [2.18–4.19] N = 3787	1.17 [0.88–1.46] N = 10 940	0.22 [0.13–0.32] N = 4831
Belgium	6.48 [5.76–7.19] N = 9110	1.23 [0.00–2.65] N = 340	4.39 [3.63–5.15] N = 6064	12.24 [10.54–13.94] N = 2706	4.71 [4.15–5.28] N = 5663	1.76 [1.31–2.21] N = 3447
France	2.44 [2.16–2.73] N = 15 481	0.61 [0.00–1.36] N = 611	1.40 [1.14–1.66] N = 10 061	5.05 [4.30–5.80] N = 4809	2.07 [1.80–2.33] N = 11 005	0.38 [0.27–0.49] N = 4476
Germany	1.29 [1.11–1.47] N = 23 241	0.40 [0.00–0.97] N = 772	0.65 [0.51–0.80] N = 15 707	2.86 [2.36–3.36] N = 6762	0.60 [0.48–0.72] N = 15 943	0.69 [0.56–0.83] N = 7298
Luxembourg	2.74 [2.20–3.28] N = 3860	2.59 [0.00–5.61] N = 115	2.71 [2.09–3.33] N = 2840	2.85 [1.67–4.03] N = 905	2.68 [2.14–3.21] N = 2607	0.06 [0.00–0.14] N = 1253
Netherlands	3.15 [2.74–3.55] N = 7652	0.28 [0.00–0.82] N = 349	2.23 [1.79–2.66] N = 4882	5.84 [4.85–6.84] N = 2421	2.82 [2.43–3.20] N = 5433	0.33 [0.19–0.47] N = 2219

The 95% CI are given between brackets. An individual was considered to have vision problems if he declared having a lot of difficulty or not being able to see at all when answering to the item ‘difficulty in seeing, even when wearing glasses or contact lenses’. An individual was considered to have no vision problem if he/she answered that they had no difficulty or some difficulty in seeing. Note that Europe includes Cyprus, which was not part of any region defined by the United Nations.

Women reported significantly more vision problems than men did with overall age‐adjusted prevalence of self‐reported vision problems of 2.41% [2.31–2.51] versus 1.70% [1.61–1.78], respectively (OR and 95% CI: 1.43 [1.34–1.54]).

Among older participants, women reported more vision problems than males, with an age‐adjusted prevalence of 5.65% [5.38–5.92] for women and 3.62% [3.40–3.84] for males in the age group of 60+ years (OR and 95% CI: 1.60 [1.47–1.74]), reaching 17.22% [15.68–18.76] for women and 11.85% [10.25–13.45] for males in the age group of 85+ years (OR and 95% CI: 1.55 [1.28–1.87]). These results are displayed in Table 3.

**Table 3:** Age‐adjusted prevalence of self‐reported vision problems by sex for older individuals.

Age	Gender	N	Prevalence (%) [95% CI]	OR (95% CI)
All	M	142 662	1.70 [1.61–1.78]	1.43 [1.34–1.54]
	F	168 724	2.41 [2.31–2.51]	
50+	M	72 285	2.9 [2.74–3.05]	1.52 [1.41–1.63]
	F	85 599	4.32 [4.13–4.51]	
60+	M	46 953	3.62 [3.40–3.84]	1.60 [1.47–1.74]
	F	60 228	5.65 [5.38–5.92]	
70+	M	23 136	5.07 [4.70–5.45]	1.67 [1.52–1.85]
	F	32 218	8.18 [7.74–8.62]	
85+	M	2677	11.85 [10.25–13.45]	1.55 [1.28–1.87]
	F	4967	17.22 [15.68–18.76]	

**Fig. 2:**
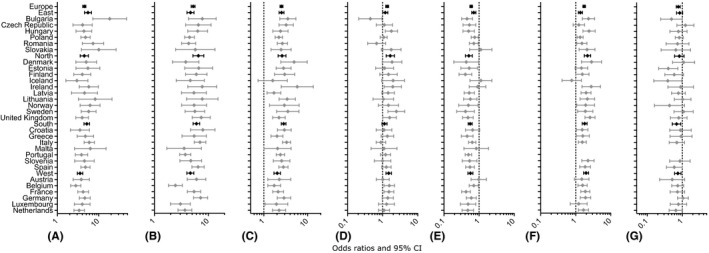
Odds ratios and 95% confidence intervals from the univariate regression analysis between vision problems and variables of interest, by region and by country, in adult population. Only variables defined from questions answered by all countries were included, namely (A) Self‐assessed health (poor versus good); (B) limiting long‐standing illness (yes versus no); (C) chronic illness (yes versus no); (D) daily smoking (yes versus no); (E) wealth (high versus low); (F) education (low versus intermediate); (G) education (high versus intermediate). The ORs of the education variable could not be computed for Malta and Portugal because no survey specified a high level of education.

The association between various factors of interest and self‐reported vision problems was investigated in adults (18 years old and older). Among other factors, depression and social isolation were associated with vision problems, with ORs of 4.55 [4.20–4.93] and 2.79 [2.43–3.21], respectively. Among those aged 65 years and more, functional limitations were associated with ORs of self‐reported vision problems of 6.04 [5.31–6.87]. These results of the univariate analysis are detailed in Table 4. Poor self‐rated health, limiting long‐standing and chronic illness, daily smoking were associated with more self‐reported vision problems with ORs of 4.48 [4.11–4.89], 5.23 [4.82–5.67], 2.53 [2.34–2.73], 1.35 [1.23–1.48], respectively, while higher wealth and education level were associated with less self‐reported vision problems, with ORs of 0.60 [0.55–0.65] and 0.77 [0.68–0.87], respectively. The results of the univariate analysis by region and countries are detailed in Fig. 2 and in supporting Information (Table S2).

**Table 4:** Univariate regression analysis between self‐reported vision problems and health, socioeconomic and lifestyle‐related variables in Europe in the adult population.

EHIS 2 (N = 302 093)			
	OR [95% CI]	% Missing values among respondents	Missing countries
Physical health			
Self‐rated health (poor versus good)	4.48 [4.11–4.89]	3.00	‐
Limiting long‐standing illness (yes versus no)	5.23 [4.82–5.67]	1.64	‐
Chronic illness (yes versus no)	2.53 [2.34–2.73]	1.39	‐
Functional limitations (yes versus no; age 65+)	6.04 [5.31–6.87]	3.62	NL, BE
Mental health			
Depression (yes versus no)	4.55 [4.20–4.93]	13.90	BE, ES, NL
Lifestyle			
Daily smoking (yes versus no)	1.35 [1.23–1.48]	1.53	
Physical activity (no versus yes)	2.26 [2.09–2.44]	9.26	BE, NL
Near‐daily alcohol consumption (yes versus no)	0.81 [0.71–0.93]	18.50	FR, IT, NL
Economics			
Wealth (higher versus low)	0.60 [0.55–0.65]	6.47	‐
Education (high versus intermediate)	0.77 [0.68–0.87]	0.69	‐
Education (low versus intermediate)	1.74 [1.61–1.89]	0.69	‐
Social life			
Social isolation (yes versus no)	2.79 [2.43–3.21]	11.72	BE, FR

‘Missing countries’ refers to those countries which did not ask one or more of the questions used to define the corresponding combined variable.

Multivariate regression analysis between self‐reported vision problems and health, socioeconomic and lifestyle‐related variables showed that limiting long‐standing illness and depression were associated with self‐reported vision problems with ORs of 2.66 [2.42–2.92] and 2.16 [2.01–2.32], respectively. Smoking, physical activity, education level, economic status and social isolation were also associated with self‐reported vision problems. These results are detailed in Table 5.

**Table 5:** Multivariate regression analysis between self‐reported vision problems and health, socioeconomic and lifestyle‐related variables in Europe in the adult population.

EHIS 2 (N = 302 093)	
	OR [95% CI]
Physical health	
Self‐rated health (poor versus good)	1.87 [1.69–2.07]
Limiting long‐standing illness (yes versus no)	2.66 [2.42–2.92]
Chronic illness (yes versus no)	1.46 [1.35–1.57]
Mental health	
Depression (yes versus no)	2.16 [2.01–2.32]
Lifestyle	
Daily smoking (yes versus no)	1.11 [1.01–1.23]
Physical activity (no versus yes)	1.31 [1.21–1.42]
Near‐daily alcohol consumption (yes versus no)	0.93 [0.80–1.08]
Economics	
Wealth (higher versus low)	0.80 [0.73–0.86]
Education (high versus intermediate)	0.95 [0.84–1.08]
Education (low versus intermediate)	1.29 [1.19–1.40]
Social life	
Social isolation (yes versus no)	1.45 [1.26–1.67]

No statistically significant association between age‐adjusted prevalence and socioeconomic indicators was found at the country level.

## Discussion

The EHIS2 population‐based survey provides data on self‐reported vision problems and associated factors for 30 countries in Europe, country by country. The crude overall prevalence of self‐reported vision problems was 2.07% [2.01–2.14].

For those aged 60 years or more, the crude prevalence of vision problems was 4.71% [4.53–4.89]. These results are slightly different from other population‐based studies of self‐reported vision status in other high‐income regions including the National Health Interview Survey (NHIS) (Lam et al. 2009), the National Health and Nutrition Examination Survey IV (NHANES IV) (Coyle et al. 2017) and the English Longitudinal Study on Ageing (ELSA) (Jackson et al. 2019). The comparison of self‐reported vision problems prevalence in these different population‐based studies is detailed in supporting Information (Table S3).

It is likely that these differing results firstly reflect the variability in the wording of visual health questions included in different surveys. Secondly, they reflect differing categorization of responses, making meaningful comparison between studies challenging. For example, in the NHIS, visual health questions were ‘Do you have any trouble seeing, even when wearing glasses or contact lenses?’ and ‘Are you blind or unable to see at all?’. Participants were classified as visually impaired if they responded yes to either question (Lam et al. 2009). In the NHANES IV, participants were asked to rate their corrected vision as excellent, good, fair, poor or very poor. Three groups were defined from the answers: poor or very poor vision, vision categorized as fair and good or excellent vision for the reference group (Coyle et al. 2017). This categorization of self‐reported vision problems was different to that which we used for EHIS 2, in which we reduced this categorization from four levels to two categories of vision problems. In ELSA, participants were asked if their corrected eyesight was excellent, very good, good, fair or poor. Respondents reporting fair or poor vision were classified in the ‘poor vision’ group (Yu et al. 2019) whereas the criteria used to define ‘vision problems’ group in the current study were more conservative. These differences are likely to explain the variability of observed prevalence of self‐reported vision problems for similar age groups. In this context, we strongly support Rein, D.B. and colleagues in advocating improved standardization of the phrasing of self‐reported vision status questions, to enhance both reproducibility and comparability of national population‐based surveys (Rein et al. 2018).

The Sustainable Development Goals (SDGs), adopted by the United Nations General Assembly in 2015, provide a new global policy framework aiming at fighting inequalities in social, economic, health and environmental aspects. Among the first five SDGs, are ‘no poverty’ (1st), ‘good health and well‐being’ (3rd), ‘quality of education’ (4th) and ‘gender equality’ (5th). Through the present analysis of the EHIS data, we were able to gain some insight into the association between the SDGs and vision impairment by the inclusion of gender, socioeconomic (income, education, social isolation and discrimination) and health data (smoking, chronic illness and functional limitation, depression).

Exploring this further, it becomes apparent that women and older respondents were more prone to report vision problems. Indeed, the age‐adjusted prevalence of self‐reported vision problems was 2.41% [2.31–2.51] for women in EHIS 2, compared to 1.70% [1.61–1.78] for males (OR and 95% CI: 1.43 [1.34–1.54]). Furthermore, when focusing on the elderly population, the age‐adjusted prevalence of vision problems was consistently higher among women than males (Table 3). In the European population, this gender difference could reflect better self‐awareness of vision impairment or less tolerance to poor vision in women, or true gender differences in the prevalence of vision impairment, relating to differences in the prevalence of underlying eye disease or to differential access to eye care services and treatments. Comparison to other studies is difficult because data on the association between gender and self‐reported vision problems are lacking. However, our results are very similar to a population‐based Canadian study, which reported that the prevalence of self‐reported uncorrected vision problems was 2.0% among women and 1.3% among males (Perruccio et al. 2010). More widely, inequality between women and males has been reported in a systematic review (Bourne et al. 2017). In that review, the authors observed that the prevalence of blindness and moderately or severely impaired vision was higher in women than in males for all age groups (0–49, 50–69 and ≥70). In line with other population‐based studies, the current study confirmed that older individuals carry a much higher risk of visual impairment. In EHIS 2, the crude prevalence of vision problems among respondents aged 70+ years was 6.88%, while the prevalence of poor vision and legal blindness was reported to be 9.08% in the 2010 Health and Retirement Study (HRS) including Americans aged 70+ years (Chen et al. 2016). In the US, the Vision and Eye Health Surveillance System (VEHSS) in the American community survey based on IRIS registry estimated that 5.60% [95% CI: 5.50–5.70] of individuals aged 65–84 years and 17% [95% CI: 16.80–17.60] of individuals aged 85+ years considered themselves to be blind or to have serious difficulties in seeing, even when wearing glasses.

Our univariate logistic regression analysis showed that even for respondents in the same country, both higher income and higher education levels were protective factors for self‐reported vision problems, with ORs of 0.60 [0.55–0.65] and of 0.77 [0.68–0.87], respectively. These results were partially confirmed in multivariate analyses which showed that higher income had a protective effect, with OR of 0.80 [0.73–0.86] while lower education level increased the risk with an OR of 1.29 [1.19–1.40].

Lower income has been frequently reported among blind or visually impaired individuals (Brézin et al. 2005). A recent study investigating the prevalence of visual impairment under the scope of socioeconomic factors at country level showed that a higher Human Development Index and Education Index were associated with a lower prevalence of blindness or moderate and severe visual impairment (Wang et al. 2017). Meanwhile, lower total health expenditure per capita and total health expenditure by Gross Domestic Product were associated with higher prevalence (Wang et al. 2017). We also analysed the relationship between socioeconomic indicators and self‐reported vision problems at the country level, but no significant association with a country’s HDI, IHDI, GDP, out of pocket expenditure, MPI and GII could be established. This may be because socioeconomic level does not differ sufficiently among member states to detect significant difference, but it is more likely that the socioeconomic associations we found at survey participant level are not reflected by the rather crude comparison of country‐level summary measures like these. Moreover, the small number of data points (30 countries) fundamentally limits the power of this analysis.

In agreement with other studies (Bourne et al. 2018), the current study also showed that self‐reported vision problems were still more prevalent in Eastern (2.43%) than in Northern (1.25%), Western (2.17%) and Southern (2.29%) European countries while the unmet needs of optical correction were the lowest in Eastern European countries (20%, see Table 2). In this context, it is likely that impact of ocular diseases on vision is more important than in other European regions. For Eastern countries, it is likely that a favourable economic evolution has not yet completely led to medical policies guaranteeing an improved access to affordable medical care. Furthermore, positive economic growth does not necessarily equate to reduced inequalities between individuals, as can be clearly observed from data on the Gini coefficient of equivalized disposable income published by EU‐SILC (https://ec.europa.eu/eurostat/web/microdata/european‐union‐statistics‐on‐income‐and‐living‐conditions). We also investigated if the payment by the national social system for eye examinations in the elderly (50+) has an impact on self‐declared vision problems. There was apparently no correlation, probably because many other factors can also interact such as the pocket‐to‐pocket expenditure for eye examination or the level of reimbursement of optical correction by social security or by insurances. In parallel to socioeconomic aspects, social isolation, a variable defined by combining celibacy and inadequate financial support, was a related risk factor for vision problems in the univariate analyses. Our cross‐sectional study also supports previous published studies which reported an association between visual impairment and depression, particularly in the elderly population (Rovner et al. 1997; Evans et al. 2007; Goldstein et al. 2012; Yip et al. 2014; Ribeiro et al. 2015; Van der Aa et al. 2015; Yu et al. 2019).

Multivariate analysis also showed that smoking status was a related risk factor for self‐reported vision problems, with an OR of 1.11 [1.01–1.25] for smokers compared with non‐smokers (Table 5). Other studies found similar results (Zhang et al. 2011).The association between smoking and vision problems could be explained by an increased risk of cataract (Kang et al. 2016) and age‐related macular degeneration (AMD) among smokers (Christen et al. 1996; Age‐Related Eye Disease Study Research Group et al. 2000; Klein et al. 2004).

We acknowledge some weaknesses in the current study. Firstly, some questions of interest were not asked in a few countries, which rendered difficult the comparison of odds ratios between different variables, and between the univariate and the multivariate analyses for the same variable. Secondly, heterogeneity between countries in the data gathering process may have been a source of measurement or selection bias, and this should be kept in mind when interpreting the results. While the prevalence of self‐reported vision problems by age group, country and gender provide a useful pan‐European insight into the epidemiology of self‐reported vision impairment, the cross‐sectional nature of the study design did not enable us to establish causal links between vision problems and explanatory variables. Thirdly, the study design of the survey did exclude people living in collective households or institutions, probably leading to an under‐estimation of self‐reported vision problems in the whole European population. Finally, the NEI‐VFQ‐25 questionnaire was not used into this survey because it was dedicated not only to vision problems, but also to wider aspects of health determinants, which are not explored with the NEI‐VFQ‐25 questionnaire. The EHIS 2 survey questionnaire was tested on population samples in different countries before being used widely on the European scale. Considering the few questions related to vision in EHIS 2, they were validated by the Washington Group on Disability Statistics short set of question that provided evidence that these questions were able to capture different aspects of difficulties in seeing.

We did not use Rasch analysis to map item responses to individual abilities, because this approach has several drawbacks. First, the resulting model would be much more difficult to interpret. Specifically, dependent variables values expressed in logits might no longer be related, even partially, to answers to questionnaire items. Moreover, the resulting effect size expressed in odds ratio in the current study could no longer be interpretable in simple terms, which would limit our results to ‘positively or negatively associated’. Second, as this approach is not currently widespread in the epidemiology community, its use would have rendered our results less accessible. There are only a few published European population‐based studies on prevalence of vision impairment and blindness (measuring visual acuity of participants rather than self‐reporting) by cause, some of them focusing on specific European countries (Munier et al. 1998; Cruciani et al. 2011; Finger et al. 2012; Havstam Johansson et al. 2020) and others having a more global focus (Flaxman et al. 2017; Bourne et al. 2018; Németh et al. 2019). According to the Vision Loss Expert Group, uncorrected refractive errors, cataract, AMD and glaucoma, that are entirely or partly curable pathologies, were still the main causes of both blindness and moderately to severely impaired vision in Western, Central and Eastern European countries (Bourne et al. 2018). Nevertheless, population‐based data on the prevalence and causes of vision problems, stratified by region and by age group, are still missing for most European Union member states. In that respect, the EHIS 2 developed and funded by the European Union represents an excellent opportunity to gather data on the health status, healthcare use and health determinants in every member state. These data, in turn, should be useful for European and local public health policies in their efforts to improve access to health services for all and to decrease inequalities (Németh et al. 2019). A strength of our study is the large size of the representative population sample, which allowed the analyses to be carried out at the level of participating countries, namely member states of Europe.

## Conclusion

This cross‐sectional European population‐based study demonstrates inequalities between European Union member states in terms of crude prevalence of self‐reported vision problems, ranging from 0.86% (in Ireland and Malta) to 6.48% (in Belgium) in the general population, with higher prevalence in Eastern European countries. Furthermore, self‐reported vision problems in Europe were more frequently observed in the elderly, women, smokers, and in those reporting greater social isolation. Higher prevalence of eye disorders in older individuals combined with other physical limitations, better self‐awareness of vision problems and economic restrictions limiting access to eye care services could explain these differences. Given that this study reports 26% of people with an unmet need for optical correction in Europe, efforts should be upscaled to address this requirement.

## Supporting information

**Table S1.** Detailed definition of the variables tested for association with visual problems.

**Table S2.** Univariate regression analysis between vision problems and health, socio‐economic and life style related variables, by region and by country, in the adult population.

**Table S3.** Prevalence of self‐reported vision problems in different population‐based studies from developed countries.
