# Supplementary material for: Self‐reported visual difficulties in Europe and related factors: a European population‐based cross‐sectional survey
Source: Acta Ophthalmol. 2020 Oct 7;99(5):559–68. doi: 10.1111/aos.14643 (PMC8451874; doi:10.1111/aos.14643)
Supplement: Supplementary file 1 — Table S1. Detailed definition of the variables tested for association with visual problems. [file AOS-99-559-s003.pdf]

EHIS2		
**Domain**	**Variables**	**Combination**
Physical Health		
Self-rated health	Self-perceived general health (HS1): Very good (1), Good (2), Fair (3), Bad (4), Very bad (5), missing (don't know, refusal) (-1)	1-2: "good health"; 3-5: "poor health"
Limiting long-standing illness	Long-standing health problem: Suffer from any illness or health problem of a duration of at least six months 1 (HS2) : yes (1), No (2), missing (don't know, refusal) (-1)<br>General activity limitation: Limitation in activities people usually do because of health problems for at least the past six months (HS3) : Severely limited (1), Limited but not severely (2), Not limited at all (3), missing (don't know, refusal) (-1)	HS2: 1: "long-standing health problem"; 2: "No l-shp"<br>HS3: 1: "severely limited"; 2: "limited but not severely"; 3: "Not limited at all"<br>**Total:**<br>If (HS2 = 1) AND (HS3 = 1-2): "Yes"<br>Else: if(HS2 = 1 AND HS3 = 3) OR HS2 = 2: "No"<br>Else: consider missing
Chronic illness	Suffering from a myocardial infarction (heart attack) in the past 12 months : yes (1), No (2), missing (don't know, refusal) (-1) (CD1c), Suffering from a coronary heart disease or angina pectoris in the past 12 months : yes (1), No (2), missing (don't know, refusal) (-1)(CD1d), Suffering from a stroke (cerebral haemorrhage, cerebral thrombosis) in the past 12 months : yes (1), No (2), missing (don't know, refusal) (CD1f), Suffering from diabetes in the past 12 months: yes (1), No (2), missing (don't know, refusal) (CD1j)	For all: 1: "Yes"; 2: "No"<br>**Total:**<br>If at least one "Yes": "Yes"<br>Else if all aquel "No": "No"<br>Else: consider missing
Vision issues	Difficulty in seeing, even when wearing glasses or contact lenses : No difficulty (1), some difficulty (2), A lot of difficulty (3), Cannot do at all/Unable to do (4), missing (don't know, refusal) (-1), not applicable (-2) (PL2)	1-2: "No"; 3-4: "Yes"

Functional limitations	Difficulty in walking half a km on level ground without the use of any aid : No difficulty (1), some difficulty (2), A lot of difficulty (3), Cannot do at all/Unable to do (4), missing (don’t know, refusal) (-1)(PL6), Difficulty in walking up or down 12 steps : No difficulty (1), some difficulty (2), A lot of difficulty (3), Cannot do at all/Unable to do (4), missing (don’t know, refusal) (-1) (PL7),<br>Difficulty in feeding yourself : No difficulty (1), some difficulty (2), A lot of difficulty (3), Cannot do at all/Unable to do (4), missing (don’t know, refusal) (-1), not applicable (-2)(PC1a), Difficulty in getting in and out of a bed or chair : No difficulty (1), some difficulty (2), A lot of difficulty (3), Cannot do at all/Unable to do (4), missing (don’t know, refusal) (-1), not applicable (-2) (PC1b), Difficulty in dressing and undressing : No difficulty (1), some difficulty (2), A lot of difficulty (3), Cannot do at all/Unable to do (4), missing (don’t know, refusal) (-1), not applicable (-2) (PC1c), Difficulty in using toilets: No difficulty (1), some difficulty (2), A lot of difficulty (3), Cannot do at all/Unable to do (4), missing (don’t know, refusal) (-1), not applicable (-2) (PC1d), Difficulty in bathing or showering : No difficulty (1), some difficulty (2), A lot of difficulty (3), Cannot do at all/Unable to do (4), missing (don’t know, refusal) (-1), not applicable (-2) (PC1e)	NB: only defined for age 65+ .<br>For all PL* and PC1*: 1: "No"; 2-4: "Yes"<br>**Total:**<br>If any "Yes": "Yes"<br>Else if all equal "No": "No"<br>Else: consider missing<br>Remark: PC2 and PC3 only get answered if at least one PC1* has been answered "Yes".
Mental health		
Depression	Suffering from depression in the past 12 months : yes (1), No (2), missing (don’t know, refusal) (-1) (CD1o),<br>Extent of having little interest or pleasure in doing things over the last 2 weeks : Not at all (1), Several days (2), More than half the days (3), Nearly every day (4), missing (don’t know, refusal) (-1)(MH1a), Extent of feeling down, depressed or hopeless over the last 2 weeks : Not at all (1), Several days (2), More than half the days (3), Nearly every day (4), missing (don’t know, refusal) (-1) (MH1b), Extent of having trouble falling or staying asleep, or sleeping too much over the last 2 weeks : Not at all (1), Several days (2), More than half the days (3), Nearly every day (4), missing (don’t know, refusal) (-1) (MH1c), Extent of feeling tired or having little energy over the last 2 weeks : Not at all (1), Several days (2), More than half the days (3), Nearly every day (4), missing (don’t know, refusal) (-1) (MH1d),	CD1o: 1: "Yes"; 2: "No"<br>For all MH1*: 1-2: "No"; 3-4: "Yes"<br>**Total:**<br>If (CD1o = 1) OR (3 or more MH1* answered "Yes"): "Yes"; Else: "No"<br>Else if (CD1o = 2) AND (3 or more MH1* answered "No"): "No"<br>Else: consider missing

	Extent of feeling bad about yourself, feeling being a failure over the last 2 weeks : Not at all (1), Several days (2), More than half the days (3), Nearly every day (4), missing (don't know, refusal) (-1) (MH1f), Extent of having trouble concentrating on things, such as reading the newspaper or watching television, over the last 2 weeks : Not at all (1), Several days (2), More than half the days (3), Nearly every day (4), missing (don't know, refusal) (-1) (MH1g)	
Lifestyle		
Physical activity	Number of days in a typical week walking to get to and from places at least 10 minutes continuously : Number of days 1 – 7, never carry out such physical activities (0), missing (don't know, refusal) (-1) (PE2), Time spent on walking to get to and from places on a typical day : 10 – 29 minutes per day (1), 30 – 59 minutes per day (2), 1 hour to less than 2 hours per day (3), 2 hours to less than 3 hours per day (4), 3 hours or more per day (5), missing (don't know, refusal) (-1), not applicable (-2) (PE3), Number of days in a typical week bicycling to get to and from places at least 10 minutes continuously : Number of days 1 – 7, never carry out such physical activities (0), missing (don't know, refusal) (-1) (PE4), Time spent on bicycling to get to and from places on a typical day : 10 – 29 minutes per day (1), 30 – 59 minutes per day (2), 1 hour to less than 2 hours per day (3), 2 hours to less than 3 hours per day (4), 3 hours or more per day (5), missing (don't know, refusal) (-1), not applicable (-2) (PE5), Number of days in a typical week doing sports, fitness or recreational (leisure) physical activities that cause at least a small increase in breathing or heart rate for at least 10 minutes continuously: Number of days 1 – 7, never carry out such physical activities (0), missing (don't know, refusal) (-1) (PE6), Time spent on doing sports, fitness or recreational (leisure) physical	PE2 > 0 AND PE3 > 1: "Yes"<br>Else if PE2 = 0 OR (PE2 > 0 AND PE3 = 1): "No"<br>Else: consider missing<br>(PE4 > 0) AND (PE5 > 1): "Yes"; else: "No"<br>Else if PE4 = 0 OR (PE4 > 0 AND PE5 = 1): "No"<br>Else: consider missing<br>(PE6 > 0) AND (PE7 >= 30 minutes): "Yes"<br>Else if PE6 = 0 OR (PE6 > 0 AND PE7 < 30 minutes): "No"<br>Else: consider missing<br>**Total:**<br>At least one "Yes": "Yes"<br>Else if all equal "No": "No"<br>Else: consider missing

	activities in a typical week : hours/minutes, missing (don't know, refusal) (-1), not applicable (-2 (PE7)	
Near-daily alcohol consumption	Frequency of consumption of an alcoholic drink of any kind (beer, wine, cider, spirits, cocktails, premixes, liqueurs, homemade alcohol…) in the past 12 months : Every day or almost (1), 5 – 6 days a week (2), 3 – 4 days a week (3), 1 – 2 days a week (4), 2 – 3 days in a month (5), Once a month (6), Less than once a month (7), Not in the past 12 months, as I no longer drink alcohol (8), Never, or only a few sips or tries, in my whole life (9), missing (don't know, refusal) (-1) (AL1)	1: "Yes"; 2-9: "No"
Daily smoking	Type of smoking behaviour : Daily smoking (1), occasional smoking (2), No smoking (3), missing (don't know, refusal) (-1) (SK1)	1: "Yes"; 2-3: "No"
Social		
In couple	Legal marital status : Never married and never been in a registered partnership (1), Married or in a registered partnership (2), Widowed or in registered partnership that ended with death of partner (not remarried or in new registered partnership) (3), Divorced or in registered partnership that was legally dissolved (not remarried or in new registered partnership) (4), missing (don't know, refusal) (-1) (MARSTALEGAL),<br>De facto marital status : Person living in a consensual union (1), Person not living in a consensual union (2), missing (don't know, refusal) (-1) (MARSTADEFACTO)	MARSTALEGAL: 2: "Yes"; 1,3,4: "No"<br>MARSTADEFACTO: 1: "Yes"; 2: "No"<br>**Total:**<br>If any "Yes": "Yes"<br>Else if all equal "No": "No"<br>Else: consider missing

Inadequate financial support	Could not afford medical examination or treatment in the past 12 months: Yes (1), No (2), No need (3), missing (don't know, refusal) (-1) (UN2a)	1: "Yes"; 2-3: "No"
Social isolation	Based on the 3 previous variables: celibacy, inadequate emotional and financial support.	If in_couple = "No" AND inadequate_financial_support = "Yes": "Yes"<br>Else if in_couple = "Yes" OR inadequate_financial_support = "No": "No"<br>Else: consider missing
Perceived discrimination		-
Economics		
Wealth quintile	Net monthly equivalised income of the household : Below 1st quintile (1), Between 1st quintile and 2nd quintile (2), Between 2nd quintile and 3rd quintile (3), Between 3rd quintile and 4th quintile (4), Between 4th quintile and 5th quintile (5), missing (don't know, refusal) (-1) (HHINCOME)	1: "low income";2-5: "Higher income"
Education	Highest level of education completed (Educational attainment) : Based on ISCED-2011 classification, Early childhood development, pre-primary education (0), Primary education (1), Lower secondary education (2), Upper secondary education (3), Post-secondary but non-tertiary education (4), Tertiary education short-cycle (5), Tertiary education, bachelor level or equivalent (6), Tertiary education, master level or equivalent (7), Tertiary education, doctoral level or equivalent (8), missing (don't know, refusal) (-1) (HATLEVEL)	0-2: "low"<br>3-5: "intermediate"<br>6-8: "high"

Table S1. Self-reported visual difficulties in Europe: definition of variables for data analysis.
