# Supplementary material for: Self‐reported visual difficulties in Europe and related factors: a European population‐based cross‐sectional survey
Source: Acta Ophthalmol. 2020 Oct 7;99(5):559–68. doi: 10.1111/aos.14643 (PMC8451874; doi:10.1111/aos.14643)
Supplement: Supplementary file 2 — Table S2. Univariate regression analysis between vision problems and health, socio‐economic and life style related variables, by region and by country, in the adult population. [file AOS-99-559-s001.pdf]

	N	Self-rated health (poor vs good)	Limiting long-standing illness (yes vs no)	Chronic illness (yes vs no)	Daily smoking (yes vs no)	Wealth (higher vs low)	Education (high vs intermediate)	Education (low vs intermediate)
**Europe**	302,093	4.48 [4.11 - 4.89]	5.23 [4.82 - 5.67]	2.53 [2.34 - 2.73]	1.35 [1.23 - 1.48]	0.60 [0.55 - 0.65]	0.77 [0.68 - 0.87]	1.74 [1.61 - 1.89]
**East**	63,158	5.51 [4.55 - 6.69]	4.66 [4.02 - 5.41]	2.54 [2.23 - 2.89]	1.19 [1.01 - 1.41]	0.71 [0.62 - 0.81]	0.84 [0.69 - 1.03]	1.36 [1.19 - 1.55]
**Bulgaria**	6,212	18.45 [7.19 - 47.32]	7.72 [4.28 - 13.94]	3.53 [2.31 - 5.38]	0.45 [0.21 - 0.95]	0.46 [0.32 - 0.65]	0.49 [0.24 - 1.00]	2.32 [1.55 - 3.47]
**Czech Republic**	6,617	4.07 [2.38 - 6.97]	6.53 [3.80 - 11.23]	3.24 [2.22 - 4.71]	1.12 [0.66 - 1.91]	0.53 [0.35 - 0.81]	1.23 [0.70 - 2.14]	1.23 [0.85 - 1.79]
**Hungary**	5,621	4.39 [2.83 - 6.81]	6.34 [4.16 - 9.68]	2.40 [1.61 - 3.58]	1.78 [1.19 - 2.66]	0.50 [0.35 - 0.73]	0.78 [0.44 - 1.37]	2.41 [1.61 - 3.60]
**Poland**	23,251	4.80 [3.71 - 6.21]	4.48 [3.64 - 5.51]	2.20 [1.81 - 2.68]	1.07 [0.86 - 1.34]	0.75 [0.60 - 0.95]	0.82 [0.63 - 1.07]	1.33 [1.09 - 1.63]
**Romania**	16,107	7.28 [4.02 - 13.20]	4.32 [3.20 - 5.82]	2.65 [2.02 - 3.47]	0.67 [0.37 - 1.22]	0.71 [0.53 - 0.95]	0.73 [0.35 - 1.55]	1.50 [1.08 - 2.10]
**Slovakia**	5,350	10.04 [3.83 - 26.33]	5.75 [2.48 - 13.32]	2.48 [1.46 - 4.20]	1.75 [0.92 - 3.35]	1.12 [0.54 - 2.33]	0.65 [0.25 - 1.72]	2.11 [1.25 - 3.55]
**North**	74,796	4.47 [3.56 - 5.61]	6.53 [5.19 - 8.23]	2.61 [2.12 - 3.21]	1.65 [1.26 - 2.16]	0.51 [0.41 - 0.64]	0.82 [0.60 - 1.12]	2.17 [1.76 - 2.67]
**Denmark**	5,347	4.98 [2.80 - 8.86]	3.80 [2.16 - 6.69]	4.90 [2.45 - 9.80]	1.85 [0.97 - 3.52]	0.43 [0.19 - 0.98]	1.09 [0.53 - 2.25]	2.82 [1.46 - 5.45]
**Estonia**	5,264	5.48 [2.81 - 10.68]	6.63 [3.66 - 12.03]	2.82 [1.90 - 4.18]	1.14 [0.63 - 2.05]	0.52 [0.30 - 0.90]	0.40 [0.21 - 0.75]	1.45 [0.96 - 2.20]
**Finland**	5,804	4.01 [2.50 - 6.43]	5.99 [3.79 - 9.46]	2.98 [1.75 - 5.08]	1.47 [0.83 - 2.61]	0.41 [0.27 - 0.62]	0.59 [0.32 - 1.08]	1.54 [0.99 - 2.40]
**Iceland**	9,493	2.96 [1.60 - 5.48]	4.62 [2.54 - 8.41]	1.60 [0.74 - 3.44]	1.99 [0.94 - 4.22]	1.13 [0.55 - 2.32]	0.39 [0.15 - 0.97]	0.77 [0.41 - 1.45]
**Ireland**	3,764	5.76 [3.39 - 9.80]	7.72 [4.16 - 14.32]	5.80 [2.50 - 13.44]	1.95 [1.11 - 3.43]	0.91 [0.53 - 1.56]	0.90 [0.39 - 2.09]	2.76 [1.51 - 5.06]
**Latvia**	5,011	4.54 [2.20 - 9.36]	5.40 [3.18 - 9.19]	1.70 [1.18 - 2.45]	1.43 [0.95 - 2.16]	0.53 [0.38 - 0.73]	0.78 [0.50 - 1.22]	1.97 [1.42 - 2.74]
**Lithuania**	6,827	8.24 [3.23 - 21.01]	7.72 [3.95 - 15.08]	3.42 [2.23 - 5.24]	1.04 [0.53 - 2.05]	0.57 [0.36 - 0.88]	1.02 [0.59 - 1.77]	2.15 [1.33 - 3.48]
**Norway**	7,842	6.17 [3.54 - 10.75]	5.44 [3.20 - 9.25]	2.94 [1.35 - 6.40]	1.46 [0.76 - 2.83]	0.50 [0.26 - 0.93]	0.43 [0.16 - 1.16]	1.92 [1.07 - 3.47]

**Sweden**	5,665	5.72 [3.78 - 8.66]	5.64 [3.70 - 8.59]	3.53 [1.96 - 6.34]	2.46 [1.41 - 4.27]	0.41 [0.23 - 0.73]	1.09 [0.58 - 2.06]	1.88 [1.14 - 3.10]
**United Kingdom**	19,779	3.96 [2.79 - 5.62]	7.38 [5.03 - 10.85]	2.22 [1.60 - 3.08]	1.58 [1.01 - 2.46]	0.48 [0.34 - 0.68]	0.79 [0.46 - 1.37]	2.56 [1.84 - 3.56]
**South**	86,726	5.14 [4.41 - 6.01]	6.01 [5.22 - 6.93]	2.84 [2.51 - 3.20]	1.13 [0.96 - 1.34]	0.56 [0.49 - 0.64]	0.69 [0.52 - 0.91]	1.80 [1.54 - 2.11]
**Croatia**	8,096	3.52 [2.07 - 5.98]	7.90 [4.69 - 13.29]	2.94 [2.04 - 4.22]	1.09 [0.66 - 1.82]	0.66 [0.41 - 1.05]	0.89 [0.44 - 1.80]	1.53 [1.05 - 2.24]
**Greece**	22,309	4.75 [3.01 - 7.51]	5.45 [3.24 - 9.18]	2.00 [1.46 - 2.74]	1.37 [0.90 - 2.06]	0.46 [0.31 - 0.67]	0.92 [0.45 - 1.89]	1.59 [1.04 - 2.45]
**Italy**	5,211	5.90 [4.44 - 7.85]	7.01 [5.47 - 8.99]	3.31 [2.68 - 4.09]	0.89 [0.65 - 1.22]	0.64 [0.51 - 0.81]	0.70 [0.42 - 1.16]	1.55 [1.20 - 2.00]
**Malta**	23,463	6.25 [2.59 - 15.08]	3.55 [1.70 - 7.43]	2.06 [1.01 - 4.20]	1.10 [0.47 - 2.57]	0.83 [0.37 - 1.86]	-*	-*
**Portugal**	3,936	3.84 [2.72 - 5.41]	3.73 [2.95 - 4.73]	2.39 [1.92 - 2.98]	1.21 [0.87 - 1.68]	0.50 [0.40 - 0.62]	-*	-*
**Slovenia**	17,759	4.52 [2.62 - 7.79]	4.72 [2.97 - 7.53]	2.55 [1.65 - 3.94]	1.00 [0.58 - 1.71]	0.54 [0.33 - 0.87]	0.86 [0.44 - 1.67]	2.24 [1.50 - 3.35]
**Spain**	5,952	4.79 [3.71 - 6.18]	6.44 [4.96 - 8.38]	2.80 [2.20 - 3.56]	1.28 [0.94 - 1.74]	0.55 [0.41 - 0.73]	0.59 [0.36 - 0.99]	1.85 [1.30 - 2.63]
**West**	72,676	3.54 [3.05 - 4.11]	4.64 [4.00 - 5.38]	2.02 [1.71 - 2.39]	1.49 [1.24 - 1.78]	0.57 [0.48 - 0.67]	0.75 [0.60 - 0.92]	1.98 [1.68 - 2.33]
**Austria**	15,519	3.78 [2.40 - 5.97]	6.03 [4.06 - 8.96]	2.26 [1.23 - 4.13]	1.01 [0.66 - 1.55]	0.98 [0.59 - 1.63]	0.52 [0.23 - 1.17]	1.49 [0.92 - 2.41]
**Belgium**	8,770	2.79 [2.10 - 3.70]	2.45 [1.83 - 3.30]	1.72 [1.23 - 2.41]	1.55 [1.10 - 2.18]	0.71 [0.53 - 0.95]	0.74 [0.50 - 1.10]	1.57 [1.16 - 2.13]
**France**	22,469	4.21 [3.12 - 5.67]	5.43 [4.08 - 7.24]	2.12 [1.60 - 2.82]	1.51 [1.10 - 2.08]	0.42 [0.32 - 0.57]	0.73 [0.45 - 1.17]	1.88 [1.40 - 2.53]
**Germany**	14,870	4.60 [3.40 - 6.23]	7.19 [5.32 - 9.72]	2.89 [2.02 - 4.12]	1.40 [0.97 - 2.02]	0.60 [0.43 - 0.83]	1.05 [0.74 - 1.48]	1.90 [1.34 - 2.68]
**Luxembourg**	3,745	3.99 [2.58 - 6.17]	3.03 [1.99 - 4.63]	1.94 [1.07 - 3.52]	1.29 [0.77 - 2.17]	0.49 [0.30 - 0.80]	0.78 [0.45 - 1.34]	1.22 [0.70 - 2.13]
**Netherlands**	7,303	3.36 [2.51 - 4.51]	3.68 [2.73 - 4.94]	2.23 [1.58 - 3.14]	1.09 [0.76 - 1.57]	0.48 [0.33 - 0.69]	0.66 [0.42 - 1.02]	1.67 [1.20 - 2.32]

Table S2. Univariate regression analysis between vision problems and health, socio-economic and life style related variables, by region and by country, in adult population. * Dashes are displayed where the ORs could not be computed because no survey for Portugal and Malta specified a high education level.
