# Supplementary material for: Self‐reported visual difficulties in Europe and related factors: a European population‐based cross‐sectional survey
Source: Acta Ophthalmol. 2020 Oct 7;99(5):559–68. doi: 10.1111/aos.14643 (PMC8451874; doi:10.1111/aos.14643)
Supplement: Supplementary file 3 — Table S3. Prevalence of self‐reported vision problems in different population‐based studies from developed countries. [file AOS-99-559-s002.pdf]

					severe VI in particular age group (%)			
	**Location**	**Age range**	**number of participants**	**Years**	**Whole cohort**	**50+**	**60+**	**65+**
**NHIS**	US	≥45 years	122,649	1999-2006	0,4	-	-	1,1
**NHANES IV**	US	≥ 2 years	8,806*	1999-2008	-	-	6,1	-
**ELSA**	England	≥ 50 years	7,677	2010-2017	11,9	11,9	-	-
**GBD**	high-income countries and Eastern and Central Europe	All ages	-	2015	2.74†	6,72	-	-
**EHIS**	Europe	≥ 15 years	311,386	2013-2015	2,07	3,64	4,71	5,58

Table S3. Comparison of self-reported vision problems prevalence in different population-based studies.

* for the 60+ age group

† Blindness + moderate and severe visual impairment
